# *catena*-Poly[[[diaqua­terbium(III)]-μ-6-carboxy­nicotinato-μ-pyridine-2,5-di­carboxyl­ato] dihydrate]

**DOI:** 10.1107/S1600536809008824

**Published:** 2009-03-19

**Authors:** Sheng Li, Fu-Li Zhang, Shou-Bin Wang, Hui-Ling Bai

**Affiliations:** a Institute of Immunology, Key Laboratory of Natural Drugs and Immunological Engineering of Henan Province, College of Medicine, Henan University, Kaifeng 475003, People’s Republic of China; b First Affiliated Hospital, Henan University, Kaifeng 475003, People’s Republic of China; c College of Chemistry and Chemical Engineering, Henan University, Kaifeng 475003, People’s Republic of China

## Abstract

The title compound, {[Tb(C_7_H_3_NO_4_)(C_7_H_4_NO_4_)(H_2_O)_2_]·2H_2_O}_*n*_, is isotypic with the analogous Tm^III^ compound [Li, Zhang, Wang & Bai (2009). *Acta Cryst*. E**65**, m411]. The Tb^III^ atom is octa­coordinated by two water mol­ecules and by four carboxyl­ate O atoms and two pyridyl N atoms from two pyridine-2,5-dicarboxyl­ate (2,5-pydc) and two 6-carboxy­nicotinate (2,5-Hpydc) ligands. The 2,5-pydc and 2,5-Hpydc ligands bridge Tb^III^ atoms, generating helical coordination polymers along [001]. An extensive network of O—H⋯O hydrogen bonds is formed between the coordination polymers and the uncoordinated water mol­ecules. The refined Flack parameter of 0.54 (2) suggests inversion twinning.

## Related literature

For the isotypic Tm^III^ compound, see Li *et al.* (2009 ▶). For other related structures, see: Huang *et al.* (2007 ▶).
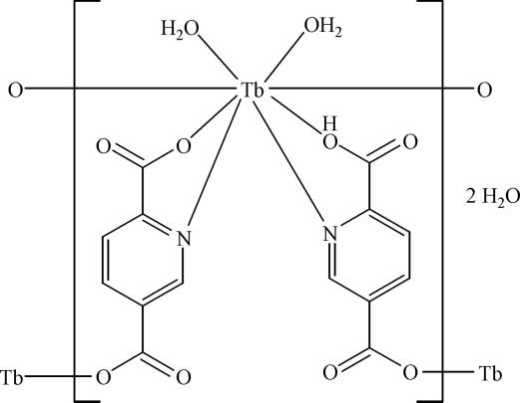


## Experimental

### 

#### Crystal data

[Tb(C_7_H_3_NO_4_)(C_7_H_4_NO_4_)(H_2_O)_2_]·2H_2_O
*M* *_r_* = 562.20
Tetragonal, 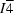

*a* = 15.107 (2) Å
*c* = 14.8587 (15) Å
*V* = 3391.1 (7) Å^3^
*Z* = 8
Mo *K*α radiation
μ = 4.25 mm^−1^
*T* = 298 K
0.12 × 0.11 × 0.08 mm

#### Data collection

Bruker APEXII CCD diffractometer
Absorption correction: multi-scan (*SADABS*; Bruker, 2001 ▶) *T*_min_ = 0.617, *T*_max_ = 0.713
6901 measured reflections
3001 independent reflections
2886 reflections with *I* > 2σ(*I*)
*R*_int_ = 0.072

#### Refinement

*R*[*F* ^2^ > 2σ(*F* ^2^)] = 0.053
*wR*(*F* ^2^) = 0.131
*S* = 1.03
3001 reflections
263 parameters
H-atom parameters constrained
Δρ_max_ = 3.51 e Å^−3^
Δρ_min_ = −1.17 e Å^−3^
Absolute structure: Flack (1983 ▶), with 1387 Friedel pairs
Flack parameter: 0.54 (2)

### 

Data collection: *APEX2* (Bruker, 2004 ▶); cell refinement: *SAINT-Plus* (Bruker, 2001 ▶); data reduction: *SAINT-Plus*; program(s) used to solve structure: *SHELXS97* (Sheldrick, 2008 ▶); program(s) used to refine structure: *SHELXL97* (Sheldrick, 2008 ▶); molecular graphics: *SHELXTL* (Sheldrick, 2008 ▶); software used to prepare material for publication: *SHELXTL*.

## Supplementary Material

Crystal structure: contains datablocks global, I. DOI: 10.1107/S1600536809008824/bi2349sup1.cif

Structure factors: contains datablocks I. DOI: 10.1107/S1600536809008824/bi2349Isup2.hkl

Additional supplementary materials:  crystallographic information; 3D view; checkCIF report

## Figures and Tables

**Table 1:** Hydrogen-bond geometry (Å, °)

*D*—H⋯*A*	*D*—H	H⋯*A*	*D*⋯*A*	*D*—H⋯*A*
O1—H1⋯O12^i^	0.85	1.98	2.801 (12)	162
O9—H91⋯O4^ii^	0.85	1.86	2.706 (10)	180
O9—H92⋯O4^iii^	0.85	1.99	2.842 (11)	180
O10—H101⋯O7^iv^	0.85	1.83	2.679 (11)	179
O10—H102⋯O9^i^	0.85	2.15	3.000 (11)	180
O11—H111⋯O5	0.85	2.00	2.851 (12)	180
O11—H112⋯O2^iv^	0.85	1.91	2.758 (12)	180
O12—H121⋯O6^v^	0.85	2.15	3.000 (12)	180
O12—H122⋯O6^vi^	0.85	2.08	2.930 (13)	180

Symmetry codes: (i) 
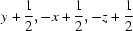
; (ii) 
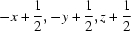
; (iii) 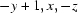; (iv) 
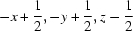
; (v) 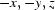; (vi) 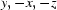.

**Fig. 1.**
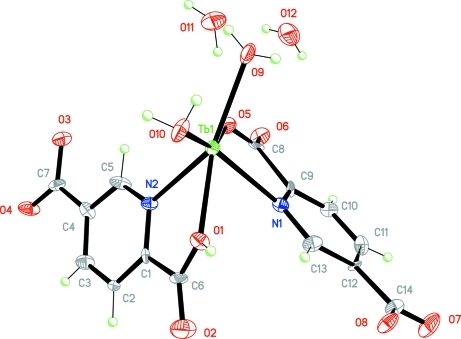
Asymmetric unit of the title compound, showing 50% probability displacement ellipsoids for non-H atoms.

**Fig. 2.**
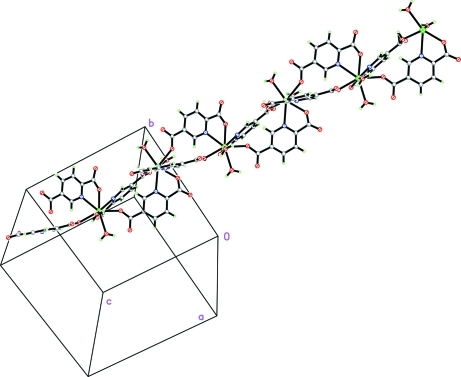
One-dimensional coordination polymer running along [001].

**Fig. 3.**
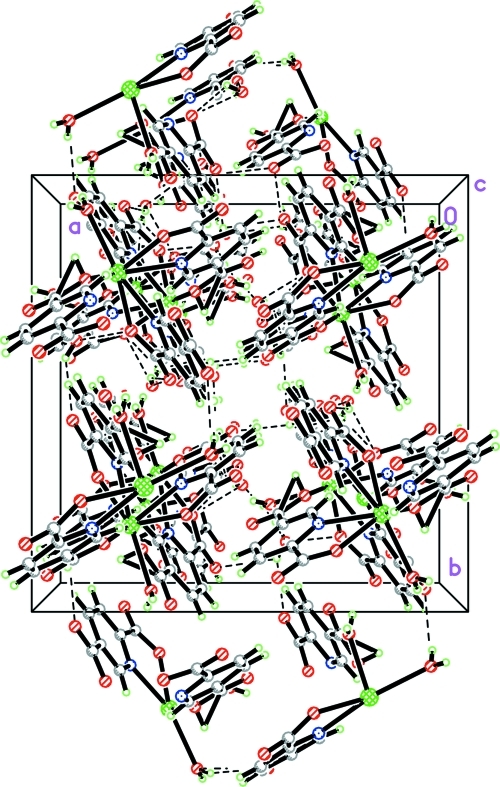
Projection along [001], showing the tetragonal arrangement of coordination polymers. O—H···O hydrogen bonds are shown as dashed lines.

## supplementary crystallographic information

### Comment

The asymmetric unit of the title compound is shown in Fig. 1. Atom Tb1 displays octa-coordination through two water molecules, four carboxylate O atoms and two pyridyl N atoms from two 2,5-pydc and two 2,5-Hpydc ligands (2,5-pydc = pyridine-2,5-dicarboxylate). The 2,5-pydc and 2,5-Hpydc ligands bridge between Tb^III^ atoms to generate helical coordination polymers along [001] (Fig. 2). An extensive network of O—H···O hydrogen bonds is formed between the coordination polymers and the lattice water molecules (Table 1 and Fig. 3).

### Experimental

A mixture of terbium oxide (0.5 mmol), pyridine-2,5-dicarboxylic acid (0.5 mmol), in H_2_O (8 ml) and ethanol (8 ml) was sealed in a 25 ml Teflon-lined stainless steel autoclave and kept at 413 K for three days. Colourless crystals were obtained after cooling to room temperature with a yield of 27%. Elemental analysis calculated for C_14_H_15_N_2_TbO_12_: C 30.68, H 2.74, N 5.11%; Found: C 30.62, H 2.72, N 5.06%.

### Refinement

H atoms bound to C atoms were placed in calculated positions with C—H = 0.93 Å and refined as riding with *U*_iso_(H) = 1.2*U*_eq_(C). H atoms of the water molecules were placed so as to form a reasonable H-bond network and refined as riding with *U*_iso_(H) = 1.5*U*_eq_(O). The Flack parameter was refined as a full least-squares variable, and the refined value of 0.54 (2) suggests inversion twinning.

### Crystal data

**Table:**

[Tb(C_7_H_3_NO_4_)(C_7_H_4_NO_4_)(H_2_O)_2_]·2H_2_O	*D*_x_ = 2.202 Mg m^−^^3^
*M**_r_* = 562.20	Mo *K*α radiation, λ = 0.71073 Å
Tetragonal, *I*4	Cell parameters from 3001 reflections
Hall symbol: I -4	θ = 1.9–25.3°
*a* = 15.107 (2) Å	µ = 4.25 mm^−^^1^
*c* = 14.8587 (15) Å	*T* = 298 K
*V* = 3391.1 (7) Å^3^	Block, colourless
*Z* = 8	0.12 × 0.11 × 0.08 mm
*F*(000) = 2192	

### Data collection

**Table:**

Bruker APEXII CCD diffractometer	3001 independent reflections
Radiation source: fine-focus sealed tube	2886 reflections with *I* > 2σ(*I*)
graphite	*R*_int_ = 0.072
φ and ω scans	θ_max_ = 25.3°, θ_min_ = 1.9°
Absorption correction: multi-scan (*SADABS*; Bruker, 2001)	*h* = −12→18
*T*_min_ = 0.617, *T*_max_ = 0.713	*k* = −18→17
6901 measured reflections	*l* = −13→17

### Refinement

**Table:**

Refinement on *F*^2^	Secondary atom site location: difference Fourier map
Least-squares matrix: full	Hydrogen site location: inferred from neighbouring sites
*R*[*F*^2^ > 2σ(*F*^2^)] = 0.053	H-atom parameters constrained
*wR*(*F*^2^) = 0.131	*w* = 1/[σ^2^(*F*_o_^2^) + (0.1007*P*)^2^ + 0.8682*P*] where *P* = (*F*_o_^2^ + 2*F*_c_^2^)/3
*S* = 1.03	(Δ/σ)_max_ < 0.001
3001 reflections	Δρ_max_ = 3.51 e Å^−^^3^
263 parameters	Δρ_min_ = −1.17 e Å^−^^3^
0 restraints	Absolute structure: Flack (1983), 1387 Freidel pairs
Primary atom site location: structure-invariant direct methods	Flack parameter: 0.54 (2)

### Special details

**Table:**

Geometry. All esds (except the esd in the dihedral angle between two l.s. planes) are estimated using the full covariance matrix. The cell esds are taken into account individually in the estimation of esds in distances, angles and torsion angles; correlations between esds in cell parameters are only used when they are defined by crystal symmetry. An approximate (isotropic) treatment of cell esds is used for estimating esds involving l.s. planes.
Refinement. Refinement of F^2^ against ALL reflections. The weighted R-factor wR and goodness of fit S are based on F^2^, conventional R-factors R are based on F, with F set to zero for negative F^2^. The threshold expression of F^2^ > 2sigma(F^2^) is used only for calculating R-factors(gt) etc. and is not relevant to the choice of reflections for refinement. R-factors based on F^2^ are statistically about twice as large as those based on F, and R- factors based on ALL data will be even larger.

### Fractional atomic coordinates and isotropic or equivalent isotropic displacement parameters (Å^2^)

**Table:**

	*x*	*y*	*z*	*U*_iso_*/*U*_eq_	
Tb1	0.30210 (3)	0.22535 (3)	0.22736 (3)	0.01388 (17)	
C1	0.1833 (6)	0.4100 (6)	0.1946 (6)	0.0116 (19)	
C2	0.1334 (7)	0.4753 (6)	0.1553 (6)	0.0148 (19)	
H2A	0.1128	0.5222	0.1900	0.018*	
C3	0.1132 (7)	0.4720 (7)	0.0639 (7)	0.019 (2)	
H3A	0.0796	0.5166	0.0376	0.023*	
C4	0.1433 (6)	0.4021 (6)	0.0129 (7)	0.015 (2)	
C5	0.1879 (7)	0.3364 (7)	0.0575 (8)	0.021 (2)	
H5A	0.2046	0.2864	0.0251	0.025*	
C6	0.2161 (6)	0.4123 (6)	0.2921 (6)	0.015 (2)	
C7	0.1302 (7)	0.3959 (7)	−0.0870 (7)	0.017 (2)	
C8	0.1196 (7)	0.1238 (6)	0.1716 (7)	0.014 (2)	
C9	0.0975 (6)	0.1488 (6)	0.2710 (7)	0.0129 (18)	
C10	0.0233 (6)	0.1192 (7)	0.3157 (7)	0.017 (2)	
H10A	−0.0195	0.0865	0.2854	0.021*	
C11	0.0131 (7)	0.1383 (7)	0.4052 (7)	0.018 (2)	
H11A	−0.0351	0.1158	0.4366	0.022*	
C12	0.0768 (6)	0.1929 (6)	0.4506 (6)	0.0115 (18)	
C13	0.1449 (7)	0.2207 (7)	0.3980 (7)	0.019 (2)	
H13A	0.1865	0.2577	0.4249	0.023*	
C14	0.0684 (7)	0.2114 (7)	0.5505 (7)	0.018 (2)	
N1	0.1583 (5)	0.2001 (5)	0.3112 (6)	0.0142 (16)	
N2	0.2094 (5)	0.3400 (5)	0.1465 (6)	0.0142 (17)	
O1	0.2739 (5)	0.3551 (4)	0.3099 (5)	0.0178 (15)	
H1	0.3058	0.3643	0.3561	0.027*	
O2	0.1862 (6)	0.4677 (6)	0.3436 (5)	0.035 (2)	
O3	0.1625 (5)	0.3283 (5)	−0.1271 (5)	0.0212 (16)	
O4	0.0921 (6)	0.4561 (5)	−0.1264 (5)	0.0262 (18)	
O5	0.1946 (5)	0.1457 (5)	0.1459 (5)	0.0208 (16)	
O6	0.0625 (5)	0.0865 (5)	0.1277 (5)	0.0240 (17)	
O7	0.0056 (6)	0.1862 (6)	0.5922 (6)	0.033 (2)	
O8	0.1326 (5)	0.2550 (5)	0.5846 (5)	0.0214 (16)	
O9	0.3704 (5)	0.0819 (5)	0.1997 (5)	0.0240 (17)	
H91	0.3822	0.0700	0.2543	0.036*	
H92	0.4222	0.0849	0.1778	0.036*	
O10	0.4504 (5)	0.2736 (5)	0.2620 (5)	0.0236 (16)	
H101	0.4641	0.2868	0.2082	0.035*	
H102	0.4875	0.2327	0.2729	0.035*	
O11	0.2668 (7)	0.0205 (6)	0.0225 (6)	0.045 (2)	
H111	0.2452	0.0578	0.0593	0.068*	
H112	0.2812	0.0241	−0.0327	0.068*	
O12	0.1257 (7)	−0.0901 (6)	0.0634 (7)	0.046 (2)	
H121	0.0724	−0.0890	0.0818	0.068*	
H122	0.1145	−0.0821	0.0079	0.068*	

### Atomic displacement parameters (Å^2^)

**Table:**

	*U*^11^	*U*^22^	*U*^33^	*U*^12^	*U*^13^	*U*^23^
Tb1	0.0145 (3)	0.0144 (3)	0.0128 (2)	0.00004 (17)	−0.00014 (19)	0.00015 (19)
C1	0.013 (5)	0.011 (4)	0.011 (4)	−0.002 (4)	0.001 (4)	0.006 (4)
C2	0.022 (5)	0.008 (4)	0.014 (4)	0.002 (4)	0.007 (4)	−0.001 (4)
C3	0.015 (5)	0.020 (5)	0.023 (5)	0.007 (4)	−0.002 (4)	−0.001 (4)
C4	0.017 (5)	0.013 (5)	0.016 (5)	−0.003 (4)	−0.011 (4)	0.000 (4)
C5	0.021 (5)	0.016 (5)	0.026 (5)	0.005 (4)	−0.007 (4)	−0.009 (4)
C6	0.016 (5)	0.012 (4)	0.017 (6)	−0.001 (4)	0.002 (4)	−0.008 (4)
C7	0.012 (5)	0.020 (5)	0.019 (5)	0.000 (4)	0.004 (4)	−0.007 (4)
C8	0.023 (5)	0.006 (4)	0.012 (4)	0.004 (4)	−0.003 (4)	−0.006 (4)
C9	0.018 (4)	0.013 (4)	0.008 (4)	−0.002 (4)	−0.002 (4)	0.006 (4)
C10	0.014 (5)	0.018 (5)	0.020 (5)	−0.001 (4)	−0.003 (4)	−0.004 (4)
C11	0.015 (5)	0.023 (5)	0.018 (5)	0.000 (4)	−0.007 (4)	0.007 (4)
C12	0.008 (4)	0.015 (4)	0.012 (4)	0.004 (4)	0.008 (4)	0.000 (4)
C13	0.018 (5)	0.022 (5)	0.017 (5)	0.001 (4)	−0.002 (4)	−0.006 (4)
C14	0.014 (5)	0.025 (6)	0.014 (5)	0.001 (5)	−0.003 (4)	−0.004 (4)
N1	0.013 (4)	0.012 (4)	0.018 (4)	−0.001 (3)	−0.001 (4)	−0.002 (4)
N2	0.013 (4)	0.015 (4)	0.015 (4)	0.003 (3)	−0.002 (3)	−0.002 (3)
O1	0.021 (3)	0.016 (3)	0.016 (3)	−0.001 (3)	−0.007 (3)	−0.003 (3)
O2	0.050 (5)	0.035 (5)	0.019 (4)	0.019 (4)	−0.006 (4)	−0.002 (4)
O3	0.020 (4)	0.026 (4)	0.017 (4)	0.007 (3)	−0.002 (3)	−0.004 (3)
O4	0.032 (4)	0.024 (4)	0.022 (4)	0.017 (3)	−0.006 (3)	−0.002 (3)
O5	0.017 (4)	0.021 (4)	0.024 (4)	−0.003 (3)	0.003 (3)	−0.004 (3)
O6	0.019 (4)	0.029 (4)	0.024 (4)	−0.005 (3)	0.001 (3)	−0.006 (3)
O7	0.030 (4)	0.045 (5)	0.024 (4)	−0.016 (4)	0.011 (4)	−0.004 (4)
O8	0.019 (4)	0.032 (4)	0.013 (3)	−0.010 (3)	0.002 (3)	−0.007 (3)
O9	0.025 (4)	0.031 (4)	0.016 (3)	0.000 (3)	0.008 (3)	0.007 (3)
O10	0.021 (4)	0.035 (4)	0.014 (4)	−0.001 (3)	0.005 (3)	0.006 (3)
O11	0.064 (6)	0.039 (5)	0.033 (5)	0.012 (5)	0.014 (5)	−0.010 (4)
O12	0.053 (6)	0.045 (6)	0.039 (5)	0.018 (5)	−0.004 (5)	0.011 (5)

### Geometric parameters (Å, °)

**Table:**

Tb1—O1	2.351 (7)	C8—C9	1.560 (14)
Tb1—O5	2.356 (7)	C9—N1	1.341 (12)
Tb1—O8^i^	2.358 (7)	C9—C10	1.379 (14)
Tb1—O3^ii^	2.371 (7)	C10—C11	1.368 (16)
Tb1—O10	2.412 (7)	C10—H10A	0.930
Tb1—O9	2.435 (8)	C11—C12	1.435 (14)
Tb1—N2	2.531 (8)	C11—H11A	0.930
Tb1—N1	2.534 (8)	C12—C13	1.359 (14)
C1—N2	1.335 (13)	C12—C14	1.516 (13)
C1—C2	1.373 (13)	C13—N1	1.343 (15)
C1—C6	1.531 (13)	C13—H13A	0.930
C2—C3	1.393 (15)	C14—O7	1.195 (14)
C2—H2A	0.930	C14—O8	1.277 (12)
C3—C4	1.378 (15)	O1—H1	0.850
C3—H3A	0.930	O3—Tb1^i^	2.371 (7)
C4—C5	1.370 (15)	O8—Tb1^ii^	2.358 (7)
C4—C7	1.500 (15)	O9—H91	0.850
C5—N2	1.363 (15)	O9—H92	0.850
C5—H5A	0.930	O10—H101	0.850
C6—O2	1.221 (13)	O10—H102	0.850
C6—O1	1.256 (12)	O11—H111	0.850
C7—O4	1.226 (13)	O11—H112	0.850
C7—O3	1.279 (13)	O12—H121	0.850
C8—O6	1.219 (12)	O12—H122	0.850
C8—O5	1.242 (13)		
			
O1—Tb1—O5	124.7 (3)	O1—C6—C1	114.1 (8)
O1—Tb1—O8^i^	116.1 (3)	O4—C7—O3	123.3 (10)
O5—Tb1—O8^i^	83.7 (2)	O4—C7—C4	119.3 (9)
O1—Tb1—O3^ii^	81.4 (3)	O3—C7—C4	117.4 (9)
O5—Tb1—O3^ii^	116.7 (3)	O6—C8—O5	127.2 (10)
O8^i^—Tb1—O3^ii^	140.3 (2)	O6—C8—C9	117.9 (9)
O1—Tb1—O10	78.8 (2)	O5—C8—C9	114.9 (8)
O5—Tb1—O10	154.8 (3)	N1—C9—C10	121.9 (10)
O8^i^—Tb1—O10	76.4 (2)	N1—C9—C8	114.6 (9)
O3^ii^—Tb1—O10	72.5 (2)	C10—C9—C8	123.5 (9)
O1—Tb1—O9	155.1 (2)	C11—C10—C9	119.4 (10)
O5—Tb1—O9	75.6 (3)	C11—C10—H10A	120.3
O8^i^—Tb1—O9	77.5 (3)	C9—C10—H10A	120.3
O3^ii^—Tb1—O9	75.8 (2)	C10—C11—C12	120.2 (10)
O10—Tb1—O9	84.9 (3)	C10—C11—H11A	119.9
O1—Tb1—N2	64.9 (3)	C12—C11—H11A	119.9
O5—Tb1—N2	74.0 (3)	C13—C12—C11	114.5 (9)
O8^i^—Tb1—N2	73.6 (3)	C13—C12—C14	124.7 (10)
O3^ii^—Tb1—N2	142.3 (3)	C11—C12—C14	120.7 (9)
O10—Tb1—N2	114.1 (3)	N1—C13—C12	126.4 (10)
O9—Tb1—N2	139.8 (3)	N1—C13—H13A	116.8
O1—Tb1—N1	73.4 (3)	C12—C13—H13A	116.8
O5—Tb1—N1	65.5 (3)	O7—C14—O8	124.1 (10)
O8^i^—Tb1—N1	145.1 (3)	O7—C14—C12	121.0 (10)
O3^ii^—Tb1—N1	72.1 (3)	O8—C14—C12	114.8 (9)
O10—Tb1—N1	137.5 (3)	C9—N1—C13	117.4 (9)
O9—Tb1—N1	108.2 (3)	C9—N1—Tb1	117.1 (7)
N2—Tb1—N1	82.1 (3)	C13—N1—Tb1	124.5 (7)
N2—C1—C2	120.3 (9)	C1—N2—C5	118.7 (9)
N2—C1—C6	115.4 (8)	C1—N2—Tb1	116.8 (6)
C2—C1—C6	124.3 (9)	C5—N2—Tb1	124.4 (7)
C1—C2—C3	120.6 (9)	C6—O1—Tb1	126.1 (6)
C1—C2—H2A	119.7	C6—O1—H1	116.9
C3—C2—H2A	119.7	Tb1—O1—H1	117.0
C4—C3—C2	119.5 (9)	C7—O3—Tb1^i^	141.6 (7)
C4—C3—H3A	120.2	C8—O5—Tb1	127.4 (6)
C2—C3—H3A	120.2	C14—O8—Tb1^ii^	137.7 (6)
C5—C4—C3	116.8 (9)	Tb1—O9—H91	96.6
C5—C4—C7	119.9 (9)	Tb1—O9—H92	114.0
C3—C4—C7	123.3 (9)	H91—O9—H92	100.6
N2—C5—C4	123.9 (9)	Tb1—O10—H101	95.5
N2—C5—H5A	118.0	Tb1—O10—H102	115.7
C4—C5—H5A	118.0	H101—O10—H102	100.9
O2—C6—O1	126.6 (9)	H111—O11—H112	132.5
O2—C6—C1	119.3 (9)	H121—O12—H122	96.9
			
N2—C1—C2—C3	−3.4 (15)	O3^ii^—Tb1—N1—C13	41.3 (8)
C6—C1—C2—C3	174.1 (9)	O10—Tb1—N1—C13	6.3 (10)
C1—C2—C3—C4	0.4 (15)	O9—Tb1—N1—C13	109.1 (8)
C2—C3—C4—C5	3.7 (15)	N2—Tb1—N1—C13	−111.0 (8)
C2—C3—C4—C7	−175.7 (10)	C2—C1—N2—C5	2.2 (14)
C3—C4—C5—N2	−5.2 (16)	C6—C1—N2—C5	−175.6 (9)
C7—C4—C5—N2	174.2 (10)	C2—C1—N2—Tb1	179.6 (7)
N2—C1—C6—O2	−170.7 (9)	C6—C1—N2—Tb1	1.9 (10)
C2—C1—C6—O2	11.7 (15)	C4—C5—N2—C1	2.3 (16)
N2—C1—C6—O1	10.2 (12)	C4—C5—N2—Tb1	−174.9 (8)
C2—C1—C6—O1	−167.4 (9)	O1—Tb1—N2—C1	−7.5 (6)
C5—C4—C7—O4	−178.2 (10)	O5—Tb1—N2—C1	134.4 (7)
C3—C4—C7—O4	1.1 (16)	O8^i^—Tb1—N2—C1	−137.6 (7)
C5—C4—C7—O3	−0.4 (15)	O3^ii^—Tb1—N2—C1	21.2 (9)
C3—C4—C7—O3	178.9 (9)	O10—Tb1—N2—C1	−71.1 (7)
O6—C8—C9—N1	−171.3 (9)	O9—Tb1—N2—C1	176.6 (6)
O5—C8—C9—N1	7.9 (12)	N1—Tb1—N2—C1	67.8 (7)
O6—C8—C9—C10	10.5 (13)	O1—Tb1—N2—C5	169.7 (9)
O5—C8—C9—C10	−170.3 (9)	O5—Tb1—N2—C5	−48.3 (8)
N1—C9—C10—C11	−3.6 (15)	O8^i^—Tb1—N2—C5	39.7 (8)
C8—C9—C10—C11	174.4 (9)	O3^ii^—Tb1—N2—C5	−161.5 (7)
C9—C10—C11—C12	3.5 (15)	O10—Tb1—N2—C5	106.2 (8)
C10—C11—C12—C13	−0.9 (14)	O9—Tb1—N2—C5	−6.1 (10)
C10—C11—C12—C14	−177.4 (9)	N1—Tb1—N2—C5	−115.0 (8)
C11—C12—C13—N1	−1.9 (15)	O2—C6—O1—Tb1	161.3 (8)
C14—C12—C13—N1	174.5 (10)	C1—C6—O1—Tb1	−19.6 (12)
C13—C12—C14—O7	178.6 (11)	O5—Tb1—O1—C6	−30.8 (9)
C11—C12—C14—O7	−5.3 (15)	O8^i^—Tb1—O1—C6	70.2 (8)
C13—C12—C14—O8	−1.4 (14)	O3^ii^—Tb1—O1—C6	−147.4 (8)
C11—C12—C14—O8	174.7 (9)	O10—Tb1—O1—C6	138.9 (8)
C10—C9—N1—C13	1.0 (14)	O9—Tb1—O1—C6	−171.0 (7)
C8—C9—N1—C13	−177.2 (8)	N2—Tb1—O1—C6	15.4 (8)
C10—C9—N1—Tb1	169.6 (7)	N1—Tb1—O1—C6	−73.5 (8)
C8—C9—N1—Tb1	−8.6 (10)	O4—C7—O3—Tb1^i^	12.3 (17)
C12—C13—N1—C9	1.8 (16)	C4—C7—O3—Tb1^i^	−165.4 (7)
C12—C13—N1—Tb1	−165.8 (8)	O6—C8—O5—Tb1	176.0 (8)
O1—Tb1—N1—C9	147.5 (7)	C9—C8—O5—Tb1	−3.1 (12)
O5—Tb1—N1—C9	5.4 (6)	O1—Tb1—O5—C8	−46.5 (9)
O8^i^—Tb1—N1—C9	35.5 (9)	O8^i^—Tb1—O5—C8	−164.1 (8)
O3^ii^—Tb1—N1—C9	−126.4 (7)	O3^ii^—Tb1—O5—C8	51.8 (8)
O10—Tb1—N1—C9	−161.3 (6)	O10—Tb1—O5—C8	157.8 (7)
O9—Tb1—N1—C9	−58.6 (7)	O9—Tb1—O5—C8	117.3 (8)
N2—Tb1—N1—C9	81.4 (7)	N2—Tb1—O5—C8	−89.3 (8)
O1—Tb1—N1—C13	−44.8 (8)	N1—Tb1—O5—C8	−0.9 (8)
O5—Tb1—N1—C13	173.0 (9)	O7—C14—O8—Tb1^ii^	23.9 (17)
O8^i^—Tb1—N1—C13	−156.8 (7)	C12—C14—O8—Tb1^ii^	−156.1 (7)

Symmetry codes: (i) −*x*+1/2, −*y*+1/2, *z*−1/2; (ii) −*x*+1/2, −*y*+1/2, *z*+1/2.

### Hydrogen-bond geometry (Å, °)

**Table:**

*D*—H···*A*	*D*—H	H···*A*	*D*···*A*	*D*—H···*A*
O1—H1···O12^iii^	0.85	1.98	2.801 (12)	162
O9—H91···O4^ii^	0.85	1.86	2.706 (10)	180
O9—H92···O4^iv^	0.85	1.99	2.842 (11)	180
O10—H101···O7^i^	0.85	1.83	2.679 (11)	179
O10—H102···O9^iii^	0.85	2.15	3.000 (11)	180
O11—H111···O5	0.85	2.00	2.851 (12)	180
O11—H112···O2^i^	0.85	1.91	2.758 (12)	180
O12—H121···O6^v^	0.85	2.15	3.000 (12)	180
O12—H122···O6^vi^	0.85	2.08	2.930 (13)	180

Symmetry codes: (iii) *y*+1/2, −*x*+1/2, −*z*+1/2; (ii) −*x*+1/2, −*y*+1/2, *z*+1/2; (iv) −*y*+1, *x*, −*z*; (i) −*x*+1/2, −*y*+1/2, *z*−1/2; (v) −*x*, −*y*, *z*; (vi) *y*, −*x*, −*z*.
